# False positive diagnosis of malignancy in a case of cryptogenic organising pneumonia presenting as a pulmonary mass with mediastinal nodes detected on fluorodeoxyglucose-positron emission tomography: a case report

**DOI:** 10.1186/1752-1947-3-124

**Published:** 2009-11-14

**Authors:** Aravind Ponnuswamy, Neeraj Mediratta, Iain D Lyburn, James P Finnerty

**Affiliations:** 1 Countess of Chester Hospital NHS Foundation Trust, Chester, Cheshire, CH2 1UL, UK; 2 Cardiothoracic Centre, Broadgreen Hospital, Liverpool, L14 3LB, UK; 3 The PETCT Department, The Cheltenham Imaging Centre, Cheltenham, Gloucestershire, GL53 7AS, UK; 4 Countess of Chester Hospital, Liverpool Road, Chester, Cheshire, CH2 1UL, UK

## Abstract

**Introduction:**

We report the case of a patient with positive findings on a lung emission tomography/computed tomography (PET/CT) scan, with possible contra lateral mediastinal involvement, which strongly suggested an inoperable lung carcinoma. The lung mass proved to be a cryptogenic organising pneumonia. While the latter has previously been shown to be PET/CT positive, mediastinal involvement simulating malignant spread has not been previously reported.

**Case presentation:**

A 50-year-old Caucasian woman presented with a history of unproductive cough and was found to have a mass in the right upper lobe as shown on chest X-ray and a computed tomography scan. A subsequent PET/CT scan showed strong uptake in the right upper lobe (maximum standard uptake values (SUVmax) 9.6) with increased uptake in the adjacent mediastinum and contralateral mediastinal nodes. Surgical resection and mediastinoscopy revealed cryptogenic organising pneumonia, with enlarged reactive mediastinal lymph nodes.

**Conclusion:**

The case illustrates the limits of PET/CT scanning as a diagnostic tool, and emphasizes the importance of obtaining histological confirmation of malignant diseases whenever possible.

## Introduction

Fluorodeoxyglucose (FDG)-positron emission tomography (PET) has an established role in diagnosing and staging malignant focal lesions and has been shown to be superior to chest computed tomography (CT) scans in assessing mediastinal involvement and distant metastases [1]. However, FDG uptake is not specific for cancer, and many reports of positive PET scans in other diseases have been published. Mycobacterial, fungal, and bacterial infections, sarcoidosis, and radiation pneumonitis have shown intense uptake on PET scan. However, tumours with low glycolytic activity such as adenomas, bronchioloalveolar carcinomas, carcinoid tumours, low grade lymphomas and sub centimeter tumour masses have revealed false negative findings on PET scans.

We present a case of a patient with a positive FDG-PET scan that was consistent with lung cancer with metastases to mediastinal nodes. The patient underwent mediastinoscopy and thoracotomy and the histological diagnosis after the operation was a cryptogenic organising pneumonia.

## Case presentation

A 50-year-old Caucasian woman presented to a chest clinic with a 1-year history of a cough. A chest X-ray performed at the time of onset of symptoms had been normal. She had a history of allergic rhinitis and had developed a wheeze, and a provisional diagnosis of allergic asthma was made by her family doctor. There was an initial improvement in her symptoms with courses of prednisolone, but by the third course she was failing to respond. She had also been given a month's trial of a proton pump inhibitor for suspected associated acid reflux. There were no complaints of loss of weight or appetite. She had smoked 20 cigarettes daily from her youth until 10 years prior to presentation.

On examination, her vital signs were normal and chest examination was clear. Spirometry revealed a forced expiratory volume in 1 second (FEV1) of 2.14 litre (100% predicted) and a forced vital capacity (FVC) of 2.65 litre (93% predicted). A chest X-ray was performed which showed ill-defined shadowing in the right upper zone. The patient subsequently developed pain over the right shoulder. A CT scan revealed a 6 × 3 cm mass in the right lung apex extending alongside the right side of the mediastinum to a level above the right hilum. A provisional diagnosis of lung cancer was made. Her case was reviewed at the lung cancer multidisciplinary team meeting and further investigations were arranged: a half body FDG-PET/CT scan, a right anterior mediastinotomy and bronchoscopy. Bronchoscopy findings were normal, and cytological analysis of a transcarinal aspiration showed a small number of lymphocytes and macrophages. Similarly, the biopsies from the mediastinotomy showed reactive lymph node tissue only.

**Figure 1:**
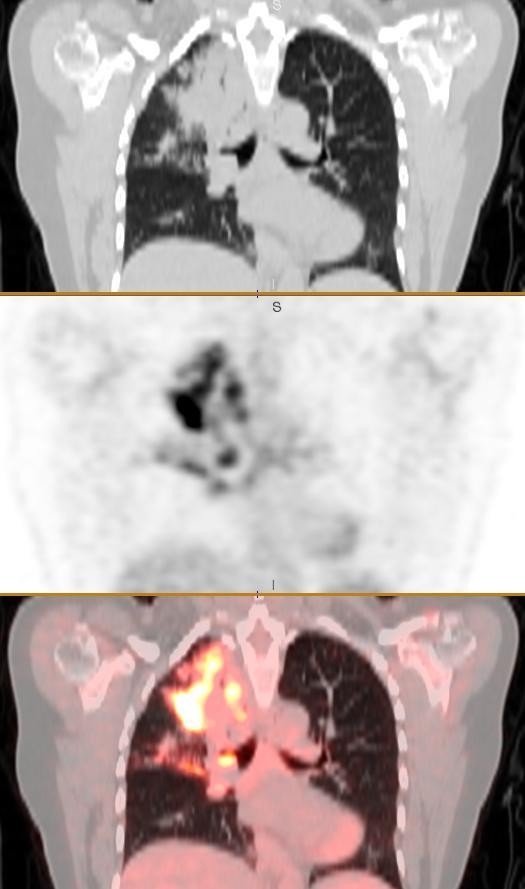
**Coronal series showing dense consolidation with areas of increased uptake in the right upper lobe: CT, positron emission tomography and fused PET/CT images**.

**Figure 2:**
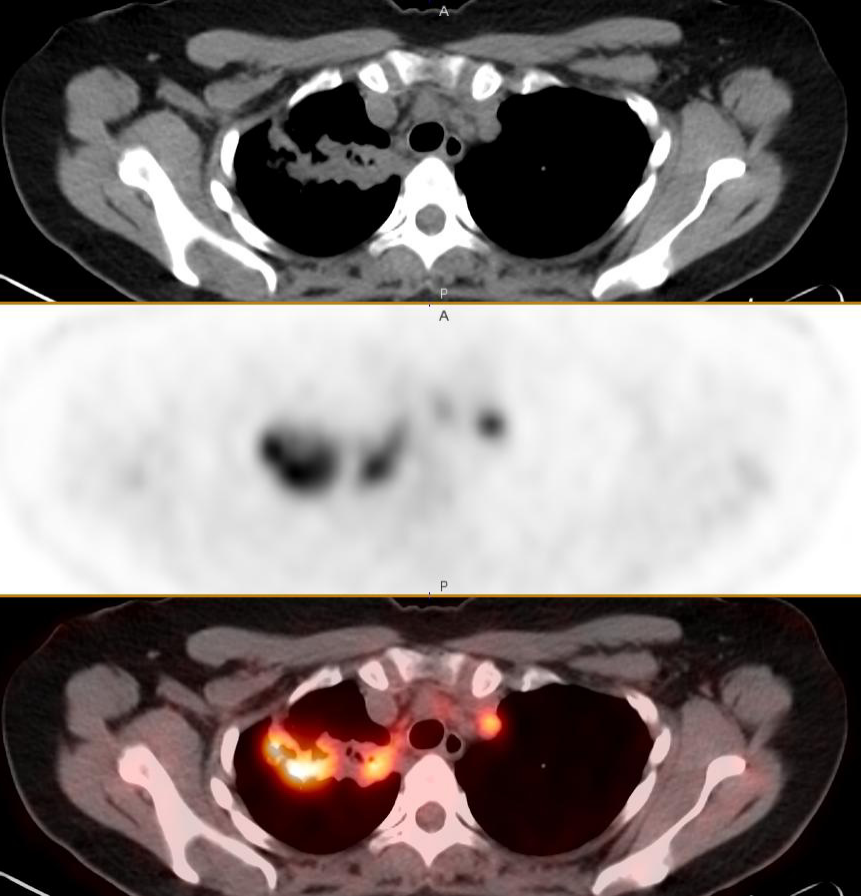
**Axial series showing dense consolidation with areas of increased uptake in the right upper lobe and focal uptake in a contralateral mediastinal node: CT, positron emission tomography and fused PET/CT images**.

The FDG-PET/CT scan showed multifocal, ill-defined semi confluent areas of marked increased uptake (maximum standard uptake values (SUVmax) of up to 9.6) within areas of dense consolidation in the right lung upper lobe (Figure 1). Focal intense uptake was present in right hilar precarinal and right and left paratracheal nodes (Figure 2). There was no abnormal uptake outside the thorax. These findings were consistent with a T4, N3 and M0 bronchial carcinoma.

Blood tests revealed eosinophilia with very high immunoglobulin E (IgE) of 1304 kAU/L (normal <87). A positive radioallergosorbent test (RAST) revealed *Aspergillus fumigates*, and *Aspergillus *precipitins were moderately positive, with a titre of 1 in 8. The eosinophil count was elevated at 0.48 × 10^9^/litre, which was 4.9% of the white cell count. The erythrocyte sedimentation rate was elevated at 64 mm/hr.

Following a multidisciplinary meeting to discuss the patient, the decision was made to proceed with surgery. Repeat bronchoscopy showed inflammation of the right main bronchus and segmental bronchi. A right posterolateral thoracotomy was performed. Adhesions were noted between the apex of the right upper lobe and the parietal pleura as well as the mediastinal pleura. Two poorly delimited tumours were evident in the right upper lobe, one towards the apex and the other in the region of the transverse fissure between the upper and middle lobes. Enlargement of the paratracheal, para-oesophageal and hilar nodes was noted. Biopsies were taken from the right upper lobe, paratracheal nodes, para-oesophageal nodes and hilar nodes. All showed reactive changes only on the frozen section. Following discussion with our oncologist in the operating theatre, and given the possibility that there was an occult neoplasm at the centre of one or both of the upper lobe masses, the upper and middle lobes were resected.

A histological examination revealed a poorly defined, tan-coloured mass, 65 mm in diameter, in the upper lobe. A microscopic examination of this mass showed organising pneumonia, with numerous macrophages and giant cells. There was no evidence of necrosis or the formation of granulomata. There was an overlying pleurisy, in which giant cells were seen. All the lymph nodes examined showed reactive changes only. Further examination and staining for *Aspergillus *did not reveal any fungi in the samples.

Two months after surgery, the patient was clinically well. In view of the positive *Aspergillus *precipitins and elevated IgE and the cryptogenic organising pneumonia on the histology, she was put on both prednisolone and itraconazole. She has had no recurrence of her respiratory symptoms since her surgery. She is currently not under therapy, with normal inflammatory markers and no evidence of consolidation on her chest X-ray 18 months after surgery.

## Discussion

There are significant limitations of the predictive value of positive PET scans, both in establishing mediastinal invasion in proven primary lung cancer and in establishing a diagnosis of lung cancer where histological proof has not been obtained. Recent guidelines on non-invasive staging of proven lung cancer gave pooled sensitivity and specificity for CT scanning and PET scanning in identifying mediastinal metastases [1]. For CT scanning, the sensitivity was 51% (95% CI: 47-54%) and specificity 85% (95% CI: 84-88%), whereas for PET/CT, the sensitivity was 74% (95% CI: 69-79%) and specificity was 85% (95% CI 82-88%). The authors emphasised that "with either test, abnormal findings must be confirmed by tissue biopsy to ensure accurate staging". A recent study looked at 100 patients whose early lung cancer was confirmed by pathology. The study found seven patients who had a false positive PET evaluation of mediastinal metastases [2], while the sensitivity for detecting involved mediastinal nodes was 87.5%. Most of the false positive results were associated with a concurrent inflammatory process. A further retrospective study of 54 patients with non-small cell lung cancer undergoing pre-operative assessment showed that for the diagnosis of mediastinal malignancy in 306 lymph nodes resected, the sensitivity of PET was 73%, and the positive predictive value of PET, that is, the percentage of truly positive nodes in those reported as positive by PET, was 70%.

High FDG uptake values tend to correlate with a greater probability of malignancy. In a multivariate analysis [3] including pathologic tumour size, involved nodes, histology, and SUVmax, only tumour size (T) of more than 3 cm and SUVmax of more than 9 and their interaction were significant predictors of survival (*P *= 0.01, 0.02, and <0.01, respectively). The 3-year survival for patients with both T <3 cm and SUVmax <9 was 97%; for those with T <3 cm and SUVmax >9, it was 94%; for those with T >3 cm and SUVmax <9, it was 93%; and for those with T >3 cm and SUVmax of >9, it was 47% (*P *< 0.01). The SUVmax of 9.6 in the lung lesion in this patient illustrates that non-malignant inflammatory lesions may have very high uptake values.

Cryptogenic organising pneumonia (sometimes known as BOOP) is well known to present, occasionally, as a solitary lesion, usually in the upper lobes and sometimes with cavitation; it is one of the mimics of lung cancer [4]. Amongst the variety of benign thoracic conditions that can demonstrate hypermetabolism on FDG-PET scans, crytogenic organising pneumonia (COP) has been reported as giving a positive FDG-PET scan in a case report [5]. COP is not commonly associated with significant enlargement of mediastinal lymph nodes: a recent study showed detectable enlargement in six out of 16 patients with COP [6]; and it is not usually associated with chest pain or pleurisy [4]. In our patient, the physical enlargement and hypermetabolism of both ipsilateral and contralateral mediastinal nodes were very unusual for COP, and led to lung resection despite the findings of only reactive changes on the frozen section.

The findings of asthma, high IgE, raised circulating eosinophils, and positive RAST and precipitin tests for *Aspergillus fumigatus *raised the possibility of allergic bronchopulmonary aspergillosis (ABPA). No evidence of fungal colonisation was found in the resected lung. Although COP has been associated with a variety of infections including fungal infections, we are not aware of any association with ABPA. The decision to treat the patient with a protracted course of itraconazole was made on the speculative grounds that fungal sensitisation may have contributed to the development of COP; however, this was not based on evidence.

## Conclusion

Our case illustrates that COP can not only simulate a lung tumour, but can also simulate inoperable lung cancer with extensive mediastinal involvement and exhibit very marked uptake on FDG-PET. It serves to reinforce the importance of obtaining a histological diagnosis of lung masses wherever possible.

## Consent

Written informed consent was obtained from the patient for publication of this case report and any accompanying images. A copy of the written consent is available for review by the Editor-in-Chief of this journal.

## Competing interests

The authors declare that they have no competing interests.

## Authors' contributions

All the authors contributed to the writing of the paper. The original manuscript was written by AP, and the final version by JPF. IDL contributed the PET/CT images and additional references.
